# An Increased Aspartate to Alanine Aminotransferase Ratio Is Associated With a Higher Risk of Cognitive Impairment

**DOI:** 10.3389/fmed.2022.780174

**Published:** 2022-04-07

**Authors:** Wei Li, Ling Yue, Lin Sun, Shifu Xiao

**Affiliations:** ^1^Department of Geriatric Psychiatry, Shanghai Mental Health Center, Shanghai Jiao Tong University School of Medicine, Shanghai, China; ^2^Alzheimer’s Disease and Related Disorders Center, Shanghai Jiao Tong University, Shanghai, China

**Keywords:** AST, ALT, hippocampal, cognitive function, elderly

## Abstract

**Background:**

Recent Alzheimer’s disease (AD) hypotheses implicate that hepatic metabolic disorders might contribute to the disease pathogenesis of AD, but the mechanism remains unclear.

**Aims:**

To investigate whether the elevated aspartate aminotransferase (AST) and Alanine aminotransferase (ALT) ratio is associated with future cognitive decline, and to explore the possible mechanisms of liver enzymes affecting cognitive function.

**Methods:**

Three different clinical cohorts were included in the current study, including one cross-sectional study (Cohort 1) and two longitudinal follow-up studies (Cohort 2 and 3). All participants completed a detailed clinical evaluation, neuropsychological tests, and liver enzyme tests. In addition, some of them also underwent structural magnetic resonance imaging (MRI) scans.

**Results:**

Cohort 1 was derived from the CRC2017ZD02 program, including 135 amnestic mild cognitive impairment (aMCI) patients, 22 AD patients, and 319 normal controls. In this cross-sectional study, we found that the AST/ALT ratio was associated with AD (*p* = 0.014, OR = 1.848, 95%CI: 1.133∼3.012), but not aMCI; Cohort 2 was derived from the Shanghai Brain Health Program. A total of 260 community elderly people with normal cognitive function were included in the study and followed up for 2 years. In this 2-year longitudinal follow-up study, we found that a higher AST/ALT ratio was a risk factor for future development of aMCI (*p* = 0.014, HR = 1.848, 95%CI: 1.133∼3.021); Cohort 3 was derived from the China longitudinal aging study (CLAS) Program. A total of 94 community elderly people with normal cognitive function were followed up for 7 years, and all of them completed MRI scans. In this 7-year longitudinal follow-up study, we found that a higher AST/ALT ratio was a risk factor for future development of aMCI (*p* = 0.006, HR = 2.247, 95%CI: 1.248∼4.049), and the AST/ALT ratio was negatively correlated with right hippocampal volume (*r* = −0.148, *p* = 0.043).

**Conclusion:**

An increased ratio of AST to ALT is associated with a higher risk of cognitive impairment and may impair cognitive function by affecting hippocampal volume.

## Introduction

Alzheimer’s disease (AD) is a progressive brain degenerative disease, which is the most common type of dementia in the world (1). At present, more than 35 million people are suffering from AD in the world, and this number is expected to double by 2030 (2). The pathological features of AD include the accumulation of tau and amyloid β as well as neuroinflammation (3). In addition, nitric oxide, inflammatory mediators, and reactive oxygen species might also contribute to the development of AD (4). There is increasing evidence that patients with Alzheimer’s disease (AD) may exhibit metabolic disorders (5), and the liver plays a critical role in the pathological process of AD (6). For example, Manivannan et al. found that the liver tissue of AD patients contained more environmental pollutants than age-matched controls (7). Giambattistelli et al. found that AD patients had significantly reduced plasma albumin levels, and prolonged prothrombin time-prothrombin time (PT) time (8). Wang et al. found that there were significant differences between AD transgenic mice and wild-type mice in the metabolite of liver and brain tissues, and multiple metabolite biomarker candidates had good identification abilities (9). Similarly, many studies have also shown that patients with cirrhotic often have changes in brain structure (10, 11). However, the mechanism by which the liver affects cognitive decline is not clear.

Alanine aminotransferase (ALT) and aspartate aminotransferase (AST) are widely used in general clinical practice to measure liver injury and have been proved to be associated with metabolic diseases, cardiovascular diseases (12), non-alcoholic fatty liver disease (NAFLD) (13), and upper tract urothelial cancer (14). Many studies have suggested that 40 U/L is the upper limit for both normal ALT and AST levels. However, these upper limits might not be adequate to exclude liver disease and to predict a risk of death from liver disease (12, 15). Weng et al. (16) found that elevated AST/ALT ratios (henceforth AST/ALT) were independently associated with an increased risk of developing cardiovascular disease (CVD) within 10 years in men. Zhou et al. (17) pointed out that the AST/ALT ratio was an independent factor in predicting the incidence of prostatic cancer (PCa). Canat et al. (18) suggested that an increased preoperative AST/ALT ratio had a significant association with renal capsule infiltration, renal vein invasion, and renal pelvis involvement in patients with non-metastatic renal cell carcinoma (RCC). However, until now, no studies have explored the plasma AST/ALT ratio and the risk of cognitive impairment. Therefore, we used three cohort studies to investigate the association between the AST/ALT ratio and cognitive impairment, and to explore the possible mechanisms by which abnormal liver function regulates cognitive function. Since AST and ALT are mainly related to Alzheimer’s disease, we mainly discuss the influence of liver enzymes on Alzheimer’s disease and its continuum (aMCI) in this study.

## Materials and Methods

### Participants

Data were obtained from the China Longitudinal Aging Study (CLAS) (19), the Brain Health Cohort study in Shanghai^1^ and the Clinical Research Center Project of Shanghai Mental Health Center (CRC2017ZD02). A total of three different cohorts were included in the current study. Ethical approval was obtained from the Ethics Committee of the Shanghai Mental Health Center, and all participants signed informed consent prior to the study.

#### Cohort 1 (the Clinical Research Center Project of Shanghai Mental Health Center)

Cohort 1 was derived from the CRC2017ZD02 program and consisted of 476 elderly people, including 135 amnestic mild cognitive impairment (aMCI) patients, 22 AD patients, and 319 normal controls. This cross-sectional study was conducted in Shanghai (China) between 2013 and 2015, and all the subjects were elderly people in the community who were not taking cognitive drugs. By using standardized questionnaires, we collected their general demographic information (such as age, gender, education, BMI), daily living information (such as smoking, drinking, drinking tea, hobbies and exercise) and disease related information (such as hypertension, diabetes, hyperlipidemia and heart disease). In addition, we also measured their liver metabolism related indicators, such as AST, ALT, high density lipoprotein, low density lipoprotein, fasting blood glucose, triglyceride, cholesterol and APOE E4.

#### Cohort 2 (Shanghai Elderly Brain Health Cohort)

Cohort 2 was derived from the Shanghai Brain Health Program. A total of 260 community elderly people with normal cognitive function were included in the study and followed up for 2 years. This project was launched in 2016, which was a prospective and observational cohort study. The specific content of this project includes understanding the mortality, prevalence, incidence, and population distribution characteristics of mild cognitive impairment and Alzheimer’s disease among the elderly over 55 years old in Shanghai communities. The inclusion criteria were as follows: (1) ≥55 years; (2) permanent population of Shanghai; (3) no evidence of serious mental illness, such as intellectual disability and schizophrenia; (4) no evidence of serious physical illness; (5) agreed to participate in the study. Exclusion criteria were as follows: (1) <55 years old; (2) floating population; (3) serious mental illness and physical illness or acute stress state, for example, acute medical disorders; and (4) the guardians or the participants or refused to participate in the study. More details could be found at the following website (see text footnote 1).

#### Cohort 3 (the China Longitudinal Aging Study)

Cohort 3 was derived from the China longitudinal aging study (CLAS) Program (19). In the current study, a total of 94 community elderly people with normal cognitive function were followed for 7 years. The biggest difference between Cohort 3 and Cohort 2 was that this cohort had T1 structural magnetic resonance (MRI) at baseline.

### Diagnostic Criteria

#### Diagnostic Criteria for Alzheimer’s Disease

The AD patients were assessed by a medical doctor specialized in dementia disorders. All participants with AD met the DSM-IV criteria for dementia as well as the NINCDS-ADRDA criteria for AD (20). All patients with AD should meet either a positive of amyloid PET scans or a positive of Aβ 42 protein in the CNS.

#### Diagnostic Criteria for Mild Cognitive Impairment Due to Alzheimer’s Disease Amnestic Mild Cognitive Impairment

The diagnosis of aMCI was based on the recommendations from the national institute on aging-Alzheimer’s association workgroups on diagnostic guidelines for Alzheimer’s disease (21): (1) concern regarding a change in cognition; (2) impairment in one or more cognitive domains; (3) preservation of independence in functional abilities; (4) not demented; (5) scored <26 points on the MoCA at the screening visit (22).

#### Diagnostic Criteria for the Normal Elderly

Subjects would be considered normal elderly if they were (1) age 55 or above; (2) scored 26–30 points on the Montreal Cognitive Assessment (MoCA) at the screening visit (22); (3) without cognitive symptoms as diagnosed by a physician; (4) without visual or hearing impairment; (5) did not meet the diagnosis of amnestic mild cognitive impairment (MCI) or dementia.

### Exclusion Criteria

Subjects should be excluded if they have the following situations: (1) under 55 years old; (2) received hormone replacement therapy; (3) took oral contraceptives; (4) with positive hepatitis B and hepatitis C viral antigens; (5) were diagnosed with fatty liver; (6) presence of an acute illness or serious mental illness (e.g., Myocardial infarction, stroke, acute infection, delirium, major depression, schizophrenia, learning disability); (7) refuse to collect plasma; (8) Misuse of alcohol or substances; (9) frontotemporal dementia, vascular dementia, and Lewy body or Parkinson dementia; (10) major depression according to geriatric depression scale (GDS 20/30) or DSM IV; (11) other diseases that might interfere with cognitive evaluation and liver function.

### Sociodemographic and Disease-Related Information

Through face-to-face interviews, we obtained the subjects’ daily life information, such as whether they smoke, drink alcohol and exercise. The way of inquiry was as follows (take smoking as an example): Do you smoke? If yes, please answer how often you smoke per week. Through their medical records and self-reports, we also obtained information about the subjects’ diseases, such as high blood pressure, diabetes, and heart disease.

### Blood Biochemical Index

All participants fasted at 10 p.m. and were tested for plasma markers the following day. Alanine aminotransferase (AST), Alanine aminotransferase (ALT), fasting plasma glucose, triglyceride, total cholesterol, high density lipoprotein, and low density lipoprotein reagents were provided by Shanghai Kehua Bio-Engineering Co., Ltd., and detection was performed using a Hitachi 7600 automatic biochemical analyzer.

### T1 Structural Magnetic Resonance

All the subjects in Cohort 3 were scanned on a 3.0-tesla MRI scanner (Siemens MAGNETOM VERIO 3.0T, Germen). The parameters of T1-weighted 3D magnetization prepared rapid gradient echo (MPRAGE) sequences were as follows: Matrix size = 240 × 256; TE = 2.98 ms; TR = 2,300 ms; flip angle of 9°; field of view (FOV) = 240 × 256 mm; slice thickness = 1.2 mm. The Learning Embedding for Atlas Propagation (LEAP) algorithm was used to ascertain volumetric data (23). The whole brain volume, hippocampus volume and amygdala volume of each individual was extracted directly using FreeSurfer (24). The selection of target brain regions, such as hippocampus and amygdala, was based on previous studies (25–27).

### Neuropsychological Tests

At baseline, all the subjects completed a battery of neuropsychological assessments which have been described previously (28), including a Chinese version of Montreal Cognitive Assessment (MoCA) (29), a Chinese version of the Rey Auditory-Verbal Learning Test (RAVLT), and a Chinese Version of Verbal Associates task. In addition, the Geriatric Depression Scale (GDS) (30) was used to exclude depression.

## Statistical Analysis

Continuous variables are expressed as mean ± standard deviation, while categorical variables are expressed as frequencies (%). The Kruskal-Wallis H (skewed distribution) test one-way ANOVA (normal distribution), and chi-square test (categorical variables) were used to determine any significant differences among the AD group, the aMCI group and the normal group. A general linear regression model was used to compare AST/ALT ratios in the AD, aMCI, and normal cognitive groups. Then logistics regression analysis was used to explore the relationship between the AST/ALT ratio and aMCI or AD (Cohort 1). In the cohort 2 and 3, we used the ROC curve was used to investigate the sensitivity and specificity of plasma AST/ASLT ratios in predicting the future occurrence of aMCI. The COX regression models were used to examine the association between the plasma AST/ALT ratio and the risk of cognitive impairment (whether to convert to aMCI as the dependent variable, the transition time was the time variable) (Cohort 2 and 3). Finally, partial correlation analysis was used to explore the association between AST/ALT ratio and brain structure, during which hypertension and exercise were controlled (Cohort 3).

## Results

### Results Associated With Cohort 1

**FIGURE 1:**
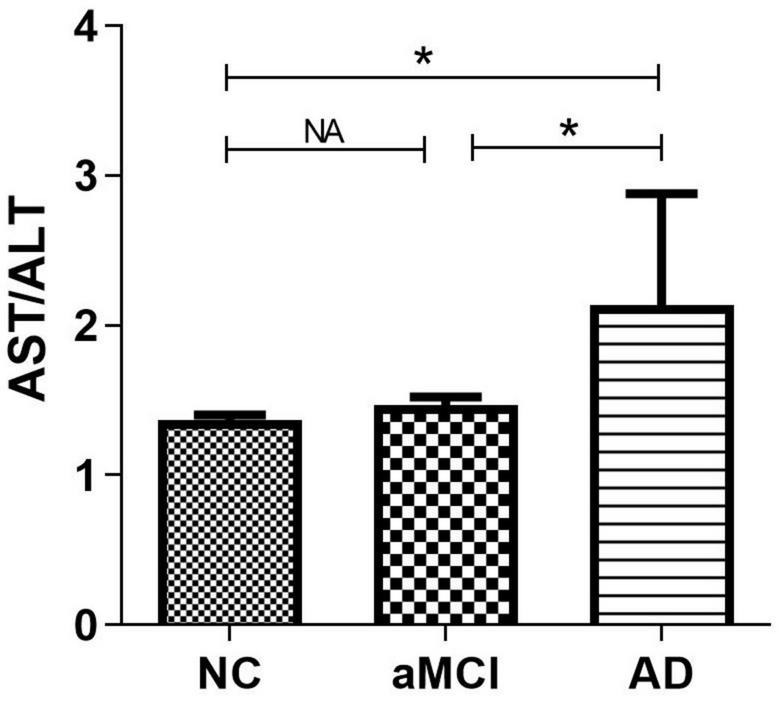
Compare the differences of AST/ALT among three groups. *p < 0.05; NA, *p* < 0.05; AST, aspartate aminotransferase; ALT, Alanine aminotransferase.

There were significant differences (*p* < 0.05) in age, education, plasma AST/ALT ratio, fasting plasma glucose, gender, smoker, drinker, hobby, MMSE scores and MoCA scores between AD, aMCI and the normal group, while there was no significant difference (*p* > 0.05) in BMI, triglyceride, cholesterol, high density lipoprotein, low density lipoprotein, take exercise, hypertension, diabetes, hyperlipidemia, heart disease, and APOE E4 among the three groups. Table 1 presents the results. By using a general linear regression model and controlling for gender, age, education, smoking, drinking alcohol, drinking tea, hobbies, and fasting blood glucose, we found that the plasma AST/ALT ratio in patients with AD was significantly higher (*p* < 0.05) than that in the normal group and the aMCI group, but there was no statistical difference (*p* > 0.05) between the normal group and the aMCI group. Figure 1 presents the results. By using multiple logistic regression analysis (no variables were controlled in the first step), we found that a higher AST/ALT ratio was associated with AD (*p* = 0.014, OR = 1.848, 95%CI: 1.133∼3.012), but not aMCI. However, when we put all the different variables into the logistics regression equation, we found no correlation between AST/ALT and AD or aMCI. Table 2 presents the results.

**TABLE 1:** Comparison of general demographic data among three groups.

Variables	aMCI	AD	Normal	*F*	*p*
	(*n* = 135)	(*n* = 22)	(*n* = 319)		
Age, y	74.93 ± 8.165	80.62 ± 5.835	69.92 ± 7.335	36.033	< 0.001*
Education, y	6.93 ± 4.918	5.35 ± 4.660	10.37 ± 3.946	38.083	< 0.001*
BMI, Kg/m^2^	23.88 ± 3.865	22.06 ± 3.091	24.03 ± 3.482	2.961	0.053
AST/ALT	1.46 ± 0.583	2.14 ± 2.682	1.37 ± 0.520	7.311	0.001*
Fasting plasma glucose, mmol/L	5.58 ± 1.790	7.14 ± 2.363	5.55 ± 1.940	3.982	0.020*
Triglyceride, mmol/L	1.64 ± 0.871	2.00 ± 1.102	1.97 ± 1.395	2.415	0.091
Cholesterol, mmol/L	4.86 ± 1.163	4.94 ± 0.730	4.83 ± 1.052	0.087	0.916
High density lipoprotein, mmol/L	1.19 ± 0.275	1.04 ± 0.244	1.14 ± 0.270	2.092	0.125
Low density lipoprotein, mmol/L	2.91 ± 0.925	2.96 ± 0.627	2.85 ± 0.838	0.246	0.782
Male, *n* (%)	45 (33.3)	3 (13.6)	152 (47.6)	15.605	< 0.001*
Smoker, *n* (%)	25 (18.5)	1 (4.5)	89 (27.9)	9.399	0.009*
Drinker, *n* (%)	21 (15.6)	0	64 (20.1)	6.328	0.042*
Tea drinker, *n* (%)	47 (34.8)	6 (27.3)	157 (49.2)	10.635	0.005*
Take exercise, *n* (%)	88 (65.2)	14 (63.6)	210 (65.8)	0.055	0.973
Hobby, *n* (%)	63 (46.7)	7 (31.8)	205 (64.3)	18.409	< 0.001*
Hypertension, *n* (%)	68 (50.4)	10 (45.5)	158 (49.5)	0.184	0.912
Diabetes, *n* (%)	19 (14.1)	5 (22.7)	43 (13.5)	1.455	0.483
Hyperlipidemia, *n* (%)	19 (14.1)	2 (9.1)	59 (18.5)	4.553	0.336
Heart disease, *n* (%)	29 (21.5)	7 (31.8)	79 (24.8)	1.296	0.523
APOE E4, *n* (%)	8 (5.9)	2 (9.1)	34 (10.7)	4.426	0.351
MMSE	23.99 ± 4.797	16.64 ± 6.426	28.19 ± 1.995	134.516	< 0.001*
moCA	17.85 ± 5.599	10.95 ± 6.358	25.24 ± 3.756	203.730	< 0.001*

ALT, Alanine transferase; AST, Glutamyl transferase; BMI, Body mass index; APOE, Apolipoprotein E; MMSE, Mini-mental State Examination; MoCA, Montreal Cognitive Assessment. *p < 0.05

**TABLE 2:** The results of logistics regression analysis.

Variables	B	S.E	Wals	Df	*p*	OR	95% confidence interval
**MCI**							
Model 1 (AST/ALT)	0.261	0.196	1.779	1	0.182	1.299	0.885∼1.907
Model 2 (AST/ALT)	0.136	0.253	0.290	1	0.590	1.146	0.689∼1.882
**AD**							
Model 1 (AST/ALT)	0.614	0.249	6.056	1	0.014*	1.848	1.133∼3.012
Model 2 (AST/ALT)	–0.392	0.628	0.390	1	0.532	0.675	0.197∼2.313

ALT, Alanine transferase; AST, Glutamyl transferase. Model 1 did not control any variables; model 2 controlled age, education, fasting plasma glucose, gender, smoker, drinker, tea drinker, and hobby. *p < 0.05.

### Results Associated With Cohort 2

**FIGURE 2:**
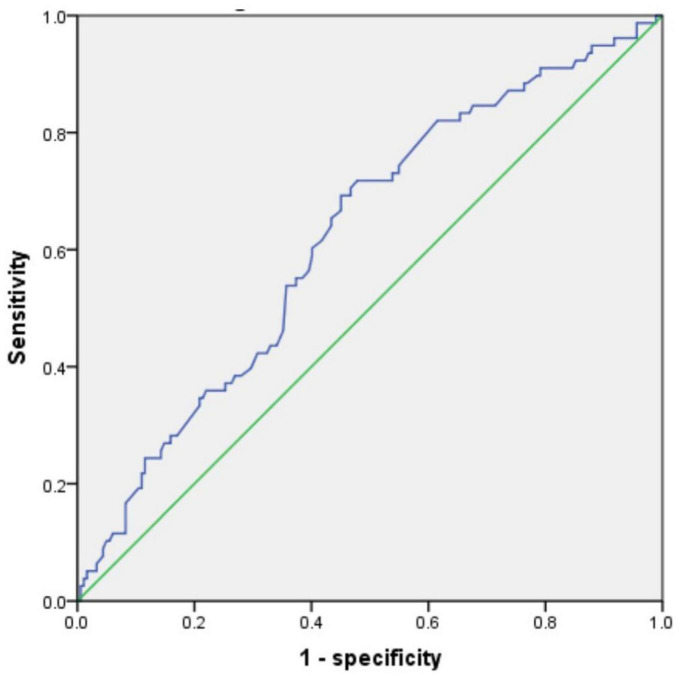
The ROC curve of AST/ALT for predicting the future occurrence of aMCI. AST, aspartate aminotransferase; ALT, Alanine aminotransferase.

**FIGURE 3:**
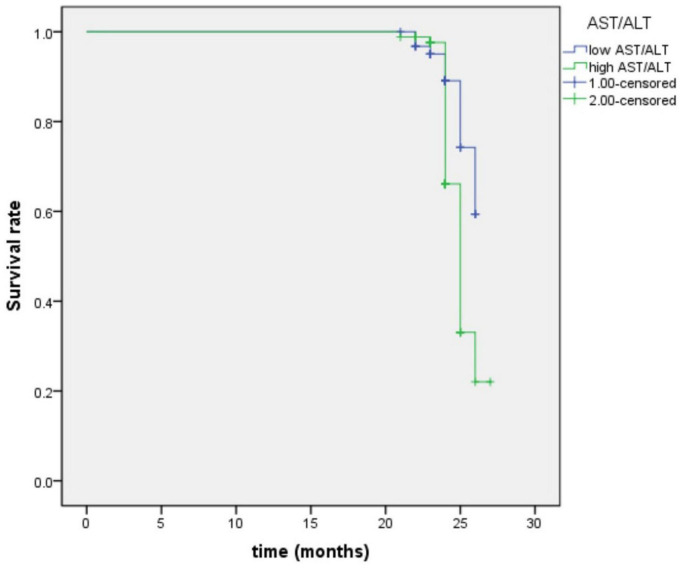
Survival regression curve of AST/ALT ratio predicting future incidence of aMCI. AST, aspartate aminotransferase; ALT, Alanine aminotransferase.

By using the ROC curve, we decided that the recommended cutoff value of AST/ALT for predicting the future occurrence of aMCI was 1.05 (sensitivity, 0.718; specificity, 0.505; the area under the ROC curve was 0.622; *p* = 0.002; 95%: 0.549∼0.695) (Figure 2). According to this cutoff value, the study population was divided into a lower AST/ALT ratio group and a higher AST/ALT ratio group. The average age of the lower AST/ALT group was significantly lower than that of the higher AST/ALT group, while the proportion of male, smokers, exercisers, fasting blood glucose and follow-up MOCA scores of the lower AST/ALT group were higher than those of the higher AST/ALT group (*p* < 0.05). There were no statistically significant differences (*p* > 0.05) in education, baseline MOCA score, alcohol intake, hypertension, diabetes, the total protein, total bilirubin, creatinine, total cholesterol, triglycerides, high density lipoprotein, or low density lipoprotein between the two groups. Table 3 presents the results. By using COX regression analysis, with future conversion to MCI as the dependent variable and transition time as the time variable, we found that high AST/ALT ratio was a risk factor for aMCI (*p* = 0.014, HR = 1.848, 95%CI: 1.133∼3.021). Table 4 and Figure 3 present the results.

**TABLE 3:** Relationship between AST/ALT and baseline demographic characteristics.

Variables	AST/ALT ≤ 1.05	AST/ALT > 1.06	X^2^ or t	*p*
	(*n* = 114)	(*n* = 146)		
Age, y	68.31 ± 7.321	71.84 ± 6.745	–4.032	< 0.001*
Education, y	11.41 ± 3.527	11.11 ± 4.109	0.627	0.531
Basline of MoCA	24.56 ± 2.727	24.03 ± 3.229	1.415	0.158
Male, *n* (%)	62 (54.4)	55 (37.7)	7.226	0.008*
Somker, *n* (%)	35 (30.7)	28 (19.2)	4.630	0.041*
Drinker, *n* (%)	30 (26.3)	31 (21.2)	0.921	0.377
Tea drinker, *n* (%)	70 (61.4)	63 (43.2)	8.536	0.004*
Take exercise, *n* (%)	87 (76.3)	93 (63.7)	4.784	0.031*
Hypertension, *n* (%)	70 (61.4)	85 (58.2)	0.270	0.613
Diabetes	33 (28.9)	31 (21.2)	2.503	0.191
The total protein, mmol/L	76.07 ± 4.817	76.90 ± 4.809	–1.379	0.169
Total bilirubin, mmol/L	14.63 ± 4.546	13.63 ± 4.682	1.747	0.082
Creatinine, mmol/L	65.24 ± 15.78	67.73 ± 18.62	–1.146	0.253
Total cholesterol, mmol/L	5.09 ± 1.080	5.13 ± 1.067	–0.313	0.754
Triglycerides, mmol/L	1.80 ± 1.019	1.73 ± 1.259	0.436	0.663
High density lipoprotein, mmol/L	1.31 ± 0.319	1.35 ± 0.294	–0.842	0.400
Low density lipoprotein, mmol/L	3.25 ± 1.005	3.16 ± 0.935	0.756	0.450
Fasting plasma glucose, mmol/L	5.83 ± 1.517	5.43 ± 1.137	2.455	0.015*
Follow-up of MOCA	24.31 ± 3.043	22.65 ± 3.670	3.887	< 0.001*

MoCA, Montreal Cognitive Assessment; ALT, Alanine transferase; AST, Glutamyl transferase. *p < 0.05.

**TABLE 4:** The results of logistics regression analysis.

Variables	B	S.E	Wals	Df	*p*	HR	95% confidence interval
AST/ALT	0.641	0.249	6.056	1	0.014*	1.848	1.133∼3.021

ALT, Alanine transferase; AST, Glutamyl transferase. *p < 0.05.

### Results Associated With Cohort 3

**FIGURE 4:**
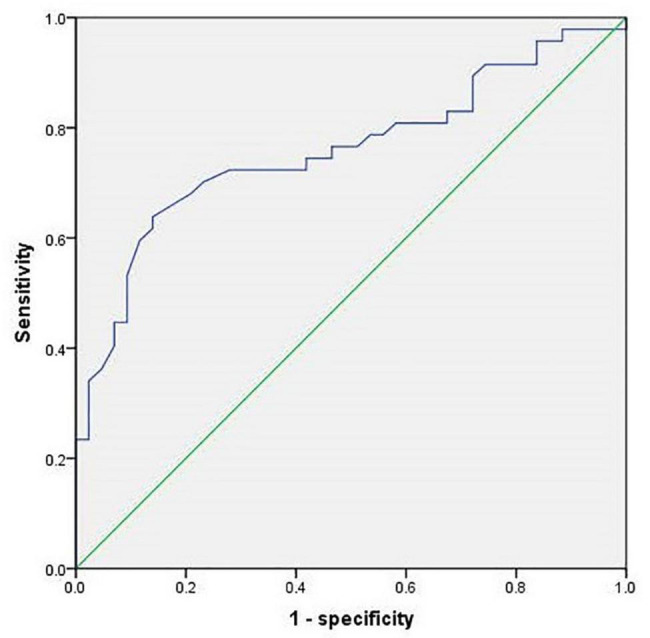
ROC curve of AST/ALT ratio to predict aMCI (cohort 3). AST, aspartate aminotransferase; ALT, Alanine aminotransferase.

**FIGURE 5:**
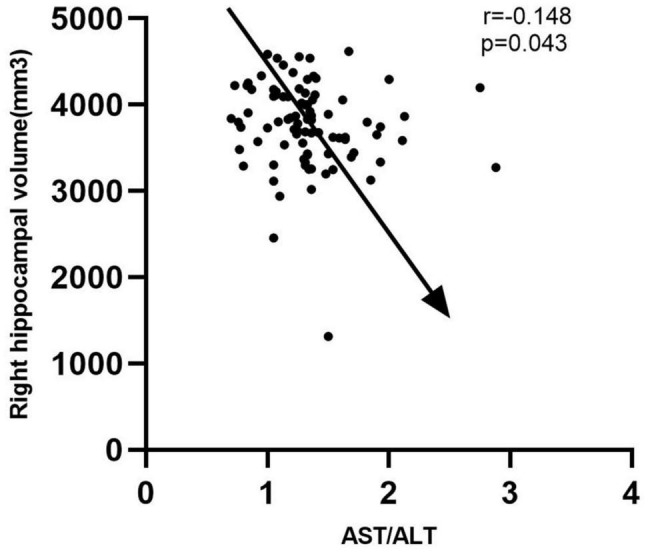
Correlation between AST/ALT ratio and right hippocampus volume. AST, aspartate aminotransferase; ALT, Alanine aminotransferase.

Based on the results of the study on cohort 2, 1.05 was still used as the threshold for AST/ALT ratio in cohort 3. The ROC curve indicated that AST/ALT predicted the area under the curve of MCI was 0.765 (*p* < 0.001; 95%: 0.610∼0.861) (Figure 4). The right baseline hippocampal volume of the low AST/ALT group was significantly larger than that of the high AST/ALT group, and the follow-up MOCA score was also significantly higher than that of the high AST/ALT group, with a lower rate of cognitive decline (*p* < 0.05), while there was no statistically significant differences (*p* > 0.05) in age, education, baseline MOCA score, gender, smoker, alcohol intake, tea drinker, take exercise, hypertension, diabetes, the total protein, total bilirubin, creatinine, total cholesterol, triglycerides, high density lipoprotein, low density lipoprotein, the whole brain volume, left hippocampus, left amygdala or right amygdala between the two groups. Table 5 presents the results. By using COX regression analysis, with future conversion to aMCI as the dependent variable and transition time as the time variable and controlled for the right baseline hippocampal volume, we found that high AST/ALT ratio was a risk factor for aMCI (*p* = 0.006, HR = 2.247, 95%CI: 1.248∼4.049) (Table 6). Correlation analysis showed that AST/ALT was negatively correlated with right hippocampal volume (*r* = −0.148, *p* = 0.043). Figure 5 presents the results.

**TABLE 5:** Relationship between AST/ALT and baseline demographic characteristics (7-years follow up).

Variables	AST/ALT ≤ 1.05	AST/ALT > 1.06	X^2^ or t	*P*
	(*n* = 42)	(*n* = 52)		
Age, y	73.10 ± 7.833	71.37 ± 6.863	1.140	0.257
Education, y	12.60 ± 5.147	12.02 ± 3.534	0.642	0.523
Basline of MoCA	22.68 ± 8.589	22.75 ± 5.216	–0.404	0.687
Male, *n* (%)	12 (28.6)	20 (38.5)	1.102	0.384
Somker, *n* (%)	6 (14.3)	8 (15.4)	0.022	1.000
Drinker, *n* (%)	5 (11.9)	11 (21.2)	1.407	0.279
Tea drinker, *n* (%)	20 (47.6)	18 (34.6)	1.631	0.214
Take exercise, *n* (%)	24 (57.1)	38 (73.1)	2.267	0.128
Hypertension, *n* (%)	22 (52.4)	31 (59.6)	0.494	0.534
Diabetes, *n* (%)	5 (11.9)	9 (17.3)	0.535	0.566
Follow-up cognitive decline, *n* (%)	14 (32.7)	37 (70.8)	14.152	< 0.001*
The total protein, mmol/L	71.82 ± 4.160	73.38 ± 5.350	–1.485	0.141
Total bilirubin,mmol/L	10.78 ± 4.181	11.89 ± 11.88	–0.557	0.579
Creatinine, mmol/L	71.37 ± 15.16	71.73 ± 18.27	–0.093	0.926
Total cholesterol, mmol/L	5.12 ± 0.909	5.05 ± 1.260	0.316	0.752
Triglycerides, mmol/L	1.95 ± 1.228	1.50 ± 0.816	1.995	0.070
High density lipoprotein, mmol/L	1.19 ± 0.227	1.29 ± 0.400	–1.515	0.134
Low density lipoprotein, mmol/L	3.18 ± 0.765	3.37 ± 1.019	–0.927	0.356
Fasting plasma glucose, mmol/L	5.46 ± 1.861	5.03 ± 0.887	1.407	0.163
Follow-up of MOCA	21.67 ± 3.989	19.19 ± 5.089	2.629	0.010*
The whole brain volume, mm^3^	1478368.65	1420594.39	1.906	0.060
Left hippocampus, mm^3^	3687.22 ± 427.73	3529.82 ± 397.59	1.849	0.068
Right hippocampus, mm^3^	3903.44 ± 471.33	3668.94 ± 520.276	2.287	0.024*
Left amygdala, mm^3^	1537.50 ± 213.47	1462.60 ± 237.98	1.604	0.112
Right amygdala, mm^3^	1667.49 ± 222.83	1594.92 ± 277.75	1.394	0.167

MoCA, Montreal Cognitive Assessment; ALT, Alanine transferase; AST, Glutamyl transferase. *p < 0.05.

**TABLE 6:** The results of logistics regression analysis (7-years follow up).

Variables	B	S.E	Wals	df	*p*	HR	95% confidence interval
AST/ALT	0.809	0.301	7.234	1	0.006*	2.247	1.248∼4.409

ALT, Alanine transferase; AST, Glutamyl transferase. *p < 0.05.

## Discussion

To my knowledge, this is the first study to examine the plasma AST/ALT ratio and the future risk of cognitive impairment, and we have come to several interesting conclusions: (1) elevated plasma AST/ALT was associated with AD but not with aMCI (Cohort 1); (2) elevated plasma AST/ALT was a risk factor for future cognitive impairment in older adults with normal baseline cognitive function (Cohort 2 and Cohort 3); (3) elevated plasma AST/ALT may contribute to cognitive impairment by affecting right hippocampal volume (Cohort 3).

About 80% of AST is present in the mitochondria of hepatocytes, while ALT is mainly present in the non-mitochondria of hepatocytes. When liver cells are damaged, AST and ALT are released from the serum, resulting in elevated serum AST and ALT levels (31). On the other hand, as liver function declines, so does the AST clearance rate (32). Therefore, serum AST level was significantly higher than serum ALT level. The ratio between the activity of AST and ALT in serum, also known as the De Ritis ratio, was first described by Fernando De Ritis in 1957. It is often used to assess liver function and reflect the severity of liver disease (33). What’s more, AST and ALT have also known to play an important role in glutamate production, which is considered as the main excitatory neurotransmitter of the central nervous system and have many critical roles in brain function.

The relationship between cognitive function and AST/ALT is extremely complex, and the mechanism is unknown. In Yoshihiro K’s study, they found that plasma AST and ALT levels were significantly negatively correlated with verbal, visual, general memory and delayed recall (34). In our study (Cohort 1), we found that the plasma AST/ALT ratio in AD patients was significantly higher than that in aMCI patients and normal controls, and was negatively correlated with the MOCA score, so our conclusions were partially consistent. To further investigate whether the plasma AST/ALT ratio could be used to predict clinical outcomes in elderly people with normal baseline cognitive function, we introduced two additional longitudinal follow-up studies (one for 2 years and the other for 7 years). The results of both cohorts (Cohort 2 and Cohort 3) suggest that an elevated AST/ALT ratio was a risk factor for future cognitive impairment. The three cohort studies mentioned above were intrinsically related, but they also had different and respective emphases, and their conclusions drawn were inconsistent. The possible heterogeneity of MCI patients might explain the discrepancies between the three studies (no differences were observed between patients without impairment and those with aMCI in the first sample, but differences were observed in the later samples). Since there were no similar longitudinal studies, we could not judge whether our findings were consistent with those of others.

Structural abnormalities in the human brain have been identified as effective biomarkers for AD, especially in its prodromal phase, and have been included in the diagnostic criteria for AD (35). The medial temporal lobe (MTL), such as the hippocampus and amygdala, are particularly interested anatomical structures in AD research, mainly because they actively participate in memory. Morphological abnormalities, including overall volume and local shape, caused by neuropathology of Alzheimer’s disease in the hippocampus and amygdala have been extensively studied (36–38). In general, such abnormalities are usually assessed by magnetic resonance imaging (MRI), such as T1-weighted imaging (39).

In the Cohort 3, we found that the right hippocampal volume of the low AST/ALT group was significantly higher than that of the high AST/ALT group, and interestingly, the AST/ALT ratio was significantly negatively correlated with the right hippocampal volume. Therefore, we proposed our study hypothesis that AST/ALT might influence the onset of cognitive impairment by influencing the volume of the right hippocampus. In Naglich A’s study (40), they found that AST/ALT was significantly associated with right and left hippocampal volume among participants aged =50. Lu et al. found that the plasma AST/ALT ratio was closely correlated with the structural change of thalamus (41). Haliloglu et al. found that AST/ALT values were significantly correlated with caudate-right lobe ratio (C/R) (42). Therefore, our conclusions were consistent.

We admit that our research has some limitations: first, the sample size was relatively small, thus limiting its reliability; second, in addition to AST and ALT, we did not include other liver metabolic indicators, such as fatty liver, etc.; third, AST and ALT were state variables that could be influenced by many other factors. During the follow-up, we only collected baseline data and could not understand the relationship between dynamic changes in liver function and cognitive function.

## Conclusion

In summary, we found that AST/ALT was a risk factor for cognitive impairment, and the mechanism might be related to the right hippocampus atrophy induced by AST/ALT.

## Data Availability Statement

The original contributions presented in the study are included in the article/supplementary material, further inquiries can be directed to the corresponding author/s.

## Ethics Statement

The studies involving human participants were reviewed and approved by the Ethics Committee of the Shanghai Mental Health Center, and all participants signed informed consent prior to the study. The patients/participants provided their written informed consent to participate in this study. Written informed consent was obtained from the individual(s) for the publication of any potentially identifiable images or data included in this article.

## Author Contributions

WL and LY contributed to the study concept and design. SX and LS analyzed the data and drafted the manuscript. All authors have read and approved the final manuscript.

## Conflict of Interest

The authors declare that the research was conducted in the absence of any commercial or financial relationships that could be construed as a potential conflict of interest.

## Publisher’s Note

All claims expressed in this article are solely those of the authors and do not necessarily represent those of their affiliated organizations, or those of the publisher, the editors and the reviewers. Any product that may be evaluated in this article, or claim that may be made by its manufacturer, is not guaranteed or endorsed by the publisher.

## Footnotes

1
http://www.shanghaibrainagingstudy.org/
